# Linguistic Traces of a Scientific Fraud: The Case of Diederik Stapel

**DOI:** 10.1371/journal.pone.0105937

**Published:** 2014-08-25

**Authors:** David M. Markowitz, Jeffrey T. Hancock

**Affiliations:** 1 Department of Communication, Cornell University, Ithaca, New York, United States of America; 2 Department of Information Science, Cornell University, Ithaca, New York, United States of America; Université de Montréal, Canada

## Abstract

When scientists report false data, does their writing style reflect their deception? In this study, we investigated the linguistic patterns of fraudulent (*N*  =  24; 170,008 words) and genuine publications (*N*  =  25; 189,705 words) first-authored by social psychologist Diederik Stapel. The analysis revealed that Stapel's fraudulent papers contained linguistic changes in science-related discourse dimensions, including more terms pertaining to methods, investigation, and certainty than his genuine papers. His writing style also matched patterns in other deceptive language, including fewer adjectives in fraudulent publications relative to genuine publications. Using differences in language dimensions we were able to classify Stapel's publications with above chance accuracy. Beyond these discourse dimensions, Stapel included fewer co-authors when reporting fake data than genuine data, although other evidentiary claims (e.g., number of references and experiments) did not differ across the two article types. This research supports recent findings that language cues vary systematically with deception, and that deception can be revealed in fraudulent scientific discourse.

## Introduction

When a scientist describes research based on fraudulent data how does his or her writing style reveal traces of their deception? The recent attention to scientific fraud [1]–[4] suggests that misconducts are on the rise across disciplines. While other work has examined statistical irregularities in fraudulent data [5], no research to date has examined linguistic distortions associated with reporting fake data. Although linguistic patterns are an indirect indicator of deception, recent research on language and psychological dynamics suggests that deceptive discourse can be distinguished from truthful discourse in a wide range of contexts [6], from interrogations [7] to fake hotel reviews [8].

Here we examine publications by social psychologist Diederik Stapel, who was found guilty of scientific fraud and whose research program has been comprehensively investigated [9]. Stapel's reports have two important attributes that lend themselves to empirical analysis. First, ground truth has been established for each publication after extensive review [9]. Second, he was highly prolific, authoring over one hundred and twenty papers, fifty-five based on fraudulent data. The investigations into Stapel's misconduct revealed, however, that he frequently allowed others to “discover” and write up the findings from data that he fraudulently generated. We therefore limit our analysis to first-authored papers, in which Stapel was most responsible for the writing, resulting in 24 fraudulent papers producing a corpus of approximately 170,008 words that we compared to a corpus of 25 genuine papers totaling 189,705 words.

Liars have difficulty approximating the appropriate frequency of linguistic dimensions for a given genre, such as the rate of spatial details in fake hotel reviews [8], the frequency of positive self-descriptions in deceptive online dating profiles [10], or the proportion of extreme positive emotions in false statements from corporate CEOs [11]. Here we investigated the frequency distributions for linguistic dimensions related to the scientific genre across the fake and genuine reports, including words related to causality (e.g., determine, impact), scientific methods (e.g., pattern, procedure), investigations (e.g., feedback, assess), and terms related to scientific reasoning (e.g., interpret, infer). We also considered language features used in describing scientific phenomena, such as quantities (e.g., multiple, enough), terms expressing the degree of relative differences (e.g., amplifiers and diminishers) and words related to certainty (e.g., explicit, certain, definite).

We were also interested in whether the fake reports contained patterns associated with deception in other contexts. Although the science genre limits the frequency of some of the most commonly observed features of deception, such as changes in the use of first-person singular pronouns (e.g. I, my) [12]–[14], there are several language dimensions that may still be relevant to investigate. Emotion terms (e.g., benefit, dislike) are often modified in deceptive language as they can reveal psychological dynamics [6], [14]. Negative affect, for example, can reflect “leakage cues” of anxiety around the deception, while positive affect can result from duping delight or a persuasion strategy to “sell” something as more desirable than it is [10], [14]–[16]. Defensiveness associated with deception can result in increased negations (e.g., nor, not) [10], [13], while discrepancy terms (e.g., could, should) serve to distract an audience from the truth [14]. Research on deception and memory reveals that explanations of fabricated events tend to be less descriptive than real events [17], suggesting that fraudulent papers should contain less detail (e.g., adjectives) than genuine papers. Finally, deceptive statements often contain less complex discourse structures than truths because of the difficulty associated with fabricating narratives. As such, we expect evidence of less complex sentences (e.g., fewer conjunctions) in fraudulent papers [12], [14].

## Method

Three committees reviewed all of Stapel's publications and issued a detailed account of his transgressions [9]. The committees established indisputable fraud in 55 publications after obtaining raw data, re-analyzing studies, and interviewing Stapel, while 70 publications were established as genuine. They report, however, no evidence of fraud by Stapel's collaborators. Our analysis therefore focuses only on Stapel's first-authored publications in which there is established evidence of fraud. The resulting corpus, after excluding papers not written in English, yielded 24 fraudulent publications (170,008 words) and 25 genuine publications (189,705 words) (see Table S1 for articles included in the analysis). Consistent with principles of scientific transparency and based on the recommendation by Simmons and colleagues [18], the fraudulent and genuine Stapel files are available from the authors.

To analyze writing style we applied a corpus analytic method using Wmatrix [19], [20], an approach that is commonly used for corpus comparisons (e.g., [21], [22]). Wmatrix is a tool that provides standard corpus linguistics analytics, including word frequency lists and analyses of major grammatical categories and semantic domains. Wmatrix tags parts of speech (e.g., adjectives, nouns) in relation to other words within the context of a sentence (e.g., the word “store” can take the noun form as a retail establishment or a verb, as the act of supplying an object for future use). Semantic content in Wmatrix is based on McArthur's Longman Lexicon of Contemporary English [23] and references 21 major discourse fields including psychological actions, states, and processes, science and technology, and language and communication (see [20] for the full semantic tagset). Wmatrix has a classification accuracy rate of 96–97% for part of speech and 92% for semantic content in English [19].

Wmatrix provides the frequency and relative percentage of words that are tagged in each corpus and computes pairwise differences based on a log-likelihood ratio (LLR) [20]. The LLR statistical measure quantifies the difference in frequency across the two corpora on the linguistic parameter of interest. In our analysis we use a conservative cut-off of *p* < .001 in order to control for multiple LLR computations (see [24]).

The fraudulent papers were collated to create one file containing all of Stapel's fraudulent writing and the genuine papers were collated into a second file containing all of his genuine writing. Only text from the main body comprising the Introduction through Discussion sections (excluding section titles, figures, tables, and legends) was included in the two corpora. In order for Wmatrix to accurately calculate word counts, symbols common to science writing (e.g., &, <, >, [,]) were replaced with characters according to Wmatrix's preprocessing guidelines [25].

## Results

### Science-related Discourse

We first examined dimensions related to scientific writing given that liars struggle to approximate the appropriate frequency of genre-related discourse [8], [10], [11]. As described in Table 1, Stapel's fraudulent writing featured significantly higher rates of terms related to scientific methods and empirical investigation compared to his genuine writing, while cause and effect terminology and quantities did not differ across the two corpora. These data suggest that fraudulent papers involved the overproduction of scientific discourse, such as terms related to explaining data and research processes.

**Table 1:** Frequencies and Percentages of Language Categories Across Stapel's Publications.

		Fraudulent		Genuine		
Discourse Category	Word Count:	170,008		189,705		
Science-related	Example	Frequency	%	Frequency	%	LLR
Means and methods	pattern, procedure	822	0.48	576	0.30	74.68****
Certainty	explicit, precise	840	0.49	646	0.34	51.13****
Investigation	feedback, research, assess	1,329	0.78	1,265	0.67	16.38****
Amplifiers	more, extreme, profoundly	1,192	0.70	1,125	0.59	16.24****
Diminishers	less, somewhat, merely	202	0.12	312	0.16	13.21***
Reasoning	interpret, comprehend	787	0.46	744	0.39	10.52†
Quantities	multiple, general, enough	703	0.41	839	0.44	1.73
Cause and effect/connection	determine, result, attribute	4,452	2.62	5,101	2.69	1.67
Deception-related						
Emotional states and processes	affective, mood	256	0.15	133	0.07	54.22****
Adjectives	cooperative, difficult	16,535	9.73	19,314	10.18	18.65****
Negations	no, not, nor	1,352	0.80	1,608	0.85	2.99
Conjunctions	and, or	5,536	3.26	6,025	3.18	1.80
Discrepancies	could, would, should	1,813	1.07	2,053	1.08	0.21

Note: Table 1 is organized by descending LLR. LLR values of 10.83 and 15.13 equate to ***p<.001 and ****p<.0001, †*p*<.01 respectively [20]. Wmatrix categories were renamed for clarity: Amplifiers  =  “Degree: Boosters,” Reasoning  =  “Understanding,” Certainty  =  “Detailed,” Discrepancies  =  “Modal Auxiliary Verbs,” and Negations  =  “Negative.”

Stapel also used words to describe comparative differences uniquely in his fraudulent articles relative to genuine articles, with more amplifying terms (e.g., extreme, exceptionally, vastly) but fewer diminishers (e.g., somewhat, partly, slightly), suggesting that Stapel linguistically enhanced his findings when reporting on fake data and avoided words that would downplay the results. Further, Stapel used more terms related to certainty in fraudulent papers, suggesting that the fraudulent papers were written with higher levels of confidence or precision when describing the results.

### Deception-related Patterns

Did the discourse in fraudulent articles display patterns similar to deception-related work from other contexts? The results here are more mixed. An important finding in deception research is lower levels of detail in deceptive relative to truthful statements [12], [17], [26], [27] and our data are consistent with this pattern. There were significantly fewer adjectives (e.g., dominant, agreeable, meaningful) in Stapel's fraudulent papers compared to genuine papers, suggesting that papers based on fake data were less descriptive overall than those based on genuine data.

Several dimensions often observed to be diagnostic in the deception literature, however, were not different across the corpora. Stapel's fraudulent publications did not contain more negations (e.g., nor, not), discrepancies (e.g., should, would, could), or fewer conjunctions (e.g., and, or, but).

Finally, consistent with other deception research, fraudulent publications used more words related to emotional actions, states and processes, suggesting that Stapel's fraudulent papers were more affect-laden. Prior work has found that liars express more negative emotions due to non-conscious leakage of anxiety [12], [14], [15]. An examination of the affect terms in Stapel's writing revealed, however, that none were related to anxiety but were instead concerned with psychological processes of the participants, such as “affect” “mood” or “emotional,” suggesting that the increased rate of affect terms in fraudulent papers was not an indicator of leakage cues for Stapel.

A second possibility is that the overproduction of affect terms was related to persuasion, perhaps using affective processes to make the findings more exciting. This would be consistent with other deception research, in which affect terms are used to exaggerate or overvalue something, such as the elegance of a hotel [8] or the attractiveness of an online dater [10]. An alternative and simpler explanation is that more of the fraudulent articles focused on affect-related topics, an important subject in social psychology. An analysis of the abstracts and keywords, however, revealed no significant difference in affect-related terms in these summaries, suggesting that the emotion effect was not due to topical differences across the corpora.

### Co-Authors, References and Reported Experiments

In addition to writing style, we examined co-author differences between Stapel's fraudulent and genuine first-authored publications. The number of authors varied significantly across article type, *t*(45)  =  2.03, *p*  = .048, with fraudulent papers having fewer authors (*M*  =  2.00, *SD*  =  0.42) than genuine papers (*M*  =  2.28, *SD*  =  0.54). We find this result even as fraudulent and genuine articles did not statistically differ in the number of experiments and references per paper. This finding is consistent with research on deception and group size [28], as it is typically easier to deceive in the presence of a smaller group than a larger one [6].

### Text Classification Accuracy

To measure the predictive success of our language features from Table 1, we used a standard leave-one-out cross validation technique across each individual publication (see Table 2). The model fit well [x^2^  =  29.30, *p*  =  .006] and accurately classified 71.4% of Stapel's papers, resulting in a significant increase above chance (51%). Given this improvement, it is tempting to consider linguistic analysis as a forensic tool for identifying fraudulent science. This does not seem feasible, at least for now, for several reasons. First, nearly thirty percent of Stapel's publications would be misclassified, with 28% of the articles incorrectly classified as fraudulent while 29% of the fraudulent articles would be missed. Second, this analysis is based only on Stapel's research program and it is unclear how models based on his discourse style would generalize to other authors or to other disciplines.

**Table 2:** Cross-Validated Classification Accuracy Across Stapel's Fraudulent and Genuine Publications.

	Predicted		
	Fraudulent	Genuine	Classification Accuracy
Fraudulent (*N* = 24)	17	7	70.8%
Genuine (*N* = 25)	7	18	72.0%
		Overall:	71.4%

## Discussion

The present study is the first to demonstrate that the deception of a fraudulent scientist is reflected in writing style. We observed significant differences in several dimensions of Stapel's writing that reflect changes in his writing style when reporting on fake data relative to genuine data. The patterns are impressive given that the only difference between the two corpora was the fact that they reported on fraudulent data. In many other respects they were identical, including each being written by the same first author and each focusing on topics within social psychology.

The most distinct change was Stapel's use of linguistic dimensions related to scientific writing in his fraudulent work. Stapel overproduced terms related to several important science genre dimensions, including words related to methods and investigation, suggesting that he had difficulty approximating the appropriate frequency of these dimensions when reporting on fake data. Although Stapel overproduced words related to methods and investigation, it was not the case that the fraudulent papers were more descriptive; in fact, he included substantially fewer adjectives in his fraudulent articles. Overall, Stapel used nearly three thousand fewer adjectives in his fake papers than in his genuine papers. This observation is consistent with deception research related to Reality Monitoring [26], [27], which asserts that descriptive recall of real experiences are more sensory and contextually driven, while recall of imagined experiences tend to reflect cognitions, rationalizations, and fewer detailed descriptions about perceptual information [6], [29]. Given that Stapel generally did not just manipulate datasets he collected, but instead fabricated them without ever collecting any information from participants, his descriptions should resemble recall of imagined experiences rather than modifications of real ones.

A second pattern related to the science genre was Stapel's use of more language to emphasize the importance and relative differences of the results, but fewer words to downplay or hedge empirical findings. In particular, we observed significantly higher rates of linguistic amplifiers that express degrees of difference but lower rates of diminishers that attenuate or equivocate descriptions of results. Stapel also wrote with more certainty when describing his fake data, using nearly one-third more certainty terms than he did in the genuine articles. Words such as “profoundly,” “extremely,” and “considerably” frame the findings as having a substantial and dramatic impact. By describing false data with words that enhanced the results, Stapel presumably attempted to emphasize the novelty and strength of his findings, which ended up being “too good to be true” [9]. This pattern of language is also consistent with other forms of deception that involve persuading readers about quality, such as fake hotel reviews that include too many superlatives relative to real reviews [8].

Our study suggests that some traditional deception indicators, negations, conjunctions and discrepancies [10], [12]–[15], were not indicative of Stapel's fraud. There are several possible reasons for why these deception patterns did not emerge here. First, the highly formalized science genre restricts some linguistic dimensions that have been observed in other deception contexts, such as first-person singular pronouns, and this may have made traditional markers of deception less relevant for the science context. Second, science writing is planned and highly edited. In contrast, most deception research involves spoken statements or conversations, in which the lies are produced extemporaneously [30]. Cues such as reduced discourse complexity, therefore, may not be important in science writing because it is produced asynchronously and with the ability to revise. Indeed, research examining financial statements written by corporate officers found that fraudulent statements tend to have more complex discourse structures, such as longer words and sentences [31], [32], rather than less complex discourse structures. Finally, our analysis considered only one author's research program. Stapel was a prolific liar and his proficiency may have attenuated any guilt or anxiety about writing false research or any cognitive challenges that may produce cues in other deception contexts.

While generalizing to other cases of scientific fraud is important, for this initial exploration into the language of scientific fraud, having a single author whose work had been closely investigated for fraud provided an important degree of control across publication types. Despite focusing on Stapel's fraud, our results also extend findings from the Levelt, Noort, and Drenth Committees [9] by showing that Stapel left traces of fraud in his writing. These traces are consistent with other work indicating that language cues are important in deception detection. It is impressive to find these patterns in scientific discourse given how often publications are edited and controlled by authorship teams. These factors suggest that looking at a broader set of authors and fraudulent research papers [2], [33] may be fruitful for illuminating deceptive scientific language across disciplines.

## Supporting Information

Table S1 **Fraudulent and Genuine Stapel Articles in the Analysis.**
(XLSX)
